# Early Prediction of Treatment Efficacy in Second-Stage Gambiense Human African Trypanosomiasis

**DOI:** 10.1371/journal.pntd.0001662

**Published:** 2012-06-05

**Authors:** Gerardo Priotto, François Chappuis, Mathieu Bastard, Laurence Flevaud, Jean-François Etard

**Affiliations:** 1 Epicentre, Paris, France; 2 Operational Centre Geneva, Médecins sans Frontiéres, Geneva University Hospitals, Geneva, Switzerland; 3 Operational Centre Barcelona-Athens, Médecins sans Frontiéres, Barcelona, Spain; 4 Institut de Recherche pour le Développement/UMI 233, Montpellier, France; Foundation for Innovative New Diagnostics (FIND), Switzerland

## Abstract

**Background:**

Human African trypanosomiasis is fatal without treatment. The long post-treatment follow-up (24 months) required to assess cure complicates patient management and is a major obstacle in the development of new therapies. We analyzed individual patient data from 12 programs conducted by Médecins Sans Frontières in Uganda, Sudan, Angola, Central African Republic, Republic of Congo and Democratic Republic of Congo searching for early efficacy indicators.

**Methodology/Principal Findings:**

Patients analyzed had confirmed second-stage disease with complete follow-up and confirmed outcome (cure or relapse), and had CSF leucocytes counts (CSFLC) performed at 6 months post-treatment. We excluded patients with uncertain efficacy outcome: incomplete follow-up, death, relapse diagnosed with CSFLC below 50/µL and no trypanosomes. We analyzed the 6-month CSFLC via receiver-operator-characteristic curves. For each cut-off value we calculated sensitivity, specificity and likelihood ratios (LR+ and LR−). We assessed the association of the optimal cut-off with the probability of relapsing via random-intercept logistic regression. We also explored two-step (6 and 12 months) composite algorithms using the CSFLC.

The most accurate cut-off to predict outcome was 10 leucocytes/µL (n = 1822, 76.2% sensitivity, 80.4% specificity, 3.89 LR+, 0.29 LR−). Multivariate analysis confirmed its association with outcome (odds ratio = 17.2). The best algorithm established cure at 6 months with < = 5 leucocytes/µL and relapse with > = 50 leucocytes/µL; patients between these values were discriminated at 12 months by a 20 leucocytes/µL cut-off (n = 2190, 87.4% sensitivity, 97.7% specificity, 37.84 LR+, 0.13 LR−).

**Conclusions/Significance:**

The 6-month CSFLC can predict outcome with some limitations. Two-step algorithms enhance the accuracy but impose 12-month follow-up for some patients. For early estimation of efficacy in clinical trials and for individual patients in the field, several options exist that can be used according to priorities.

## Author Summary

Because Human African trypanosomiasis is fatal, it is crucial for the patient to determine if curative treatment has been effective. Unfortunately this is not possible without a 24-month laboratory follow-up, which is problematic and largely unaccomplished in the field reality. Studies that assessed early indicators have used small cohorts, yielding limited statistical power plus potential bias because of including patients with equivocal outcome. We tackled this problem by pooling a large dataset which allowed for selecting cases providing strictly unequivocal information, still numerous enough to produce sound statistical evidence. We studied predictors based on the CSF leucocytes count, a laboratory technique already available in the field, evaluating their predictive power at 6 and 12 months post-treatment. We found a predictor at 6 months (10 leucocytes/µL of CSF) that has sub-optimal accuracy but may be valuable in some particular situations, plus two-step algorithms at 6 and 12 months that offer sufficient confidence to shorten the patients' follow-up. Until better biomarkers are identified, these findings represent a significant advance for this neglected disease. Benefits are foreseen both for patients and for overburdened treatment facilities. In addition, research for new treatments can be accelerated by using early predictors.

## Introduction

Human African trypanosomiasis (HAT) or sleeping sickness, caused by *Trypanosoma brucei gambiense* (most common form, West and Central Africa) and *rhodesiense* (East and Southern Africa), is fatal unless treated. After infection, the disease progresses from the easily treatable haemolymphatic first stage to the meningoencephalitic second stage, when parasites invade the central nervous system.

Patients who receive treatment can not be considered cured immediately, because the parasite may remain viable, redeveloping fully the disease many months later. A long post-treatment follow-up period is thus required to assess cure [1]. This follow-up time is fixed at 24 months by convention, although in comparative clinical trials it is considered acceptable to measure the efficacy at 18 months [2]. Follow-up consists of control visits generally every 6 months when lymph, blood and cerebrospinal fluid (CSF) are examined. The detection of trypanosomes in any body fluid unequivocally identifies a relapse. Unfortunately, parasites are often not detected early enough to allow for timely re-treatment, plus many patients do not adhere to this demanding and invasive follow-up schedule. To better detect the relapses and avert the risk for serious sequelae or death, the variation in number of white blood cells (WBC) in CSF is widely used as a proxy marker of relapse. Other markers of relapse are under investigation and not in routine field use.

Because most HAT patients are located in remote rural areas, the post-therapeutic follow-up is particularly challenging: poverty, distance, bad roads, lack of transportation, subsistence priorities, displacement (sometimes conflict related), add to the fear of the lumbar puncture. As a result, patients' compliance with follow-up decreases markedly after the first assessment at 6 months [3].

Such long follow-up is a handicap not only for routine patient management but also for therapeutic efficacy studies [4], and particularly when a sequence of clinical studies is required (e.g. dose-finding studies). Some time can be saved when a given investigational treatment is assumed to have insufficient efficacy due to early failures surpassing a pre-defined threshold. However, when the cumulative failure rate is below that threshold, the risk of subsequent final outcome (cure or relapse) can not be predicted.

Research on ways of shortening the follow-up is scarce. One study suggests that HAT patients with <5 CSF leucocytes/µL at 6 months are at low risk of relapse (negative predictive value >0.93, n = 146) [5] and that at 6 and 12 months, patients with ≥50 and ≥20CSF leucocytes/µL, respectively, are at high risk. Another study tested an algorithm combining 6 and 12 months CSF exams on a cohort of 206 treated patients showing 97.8% specificity and 94.4% sensitivity to predict relapse [6]. Considering that these promising findings originated from relatively small cohorts, recruited each time in one single centre (Bwamanda and Mbuji Mayi, DRC, respectively), and that confirmed and unconfirmed efficacy outcomes (lost to follow-up, deaths during follow-up, etc) were mixed in the assessment via assumptions, further research is needed on larger datasets and with more restrictive selection criteria.

To meet this goal, we consolidated individual-patient data from 12 sites in Uganda, Sudan, Angola, Central African Republic, Republic of Congo and Democratic Republic of Congo where Médecins Sans Frontières (MSF) had conducted HAT programs, and we selected patients with confirmed diagnosis, confirmed stage, complete follow-up (thus confirmed outcome), and meeting a restrictive, laboratory-confirmed definition of relapse, so as to maximize information certainty. Our analysis aimed at identifying early efficacy indicators using the CSF leucocytes count at 6 and 12 months after treatment.

## Methods

### Ethics statement

The study received ethical clearance from the Médecins Sans Frontières International Ethical Review Board (Geneva, Switzerland). All data analyzed were anonymized from the start.

Using a large pooled dataset from routine MSF g*ambiense* HAT control programs, we selected patients with confirmed second-stage disease and having received second-stage treatment, who completed their follow-up (minimum 22 months) until confirmation of an outcome (cured or relapsed) and who had a CSF leucocytes count performed at 6 months post-treatment. We considered 22 months as complete follow-up because in practice patients coming for control at 22–23 months are not asked to come again at 24 months.

Second stage was defined by the finding of trypanosomes in blood, lymph nodes or CSF, with ≥20 leucocytes/µL in CSF.

We excluded patients who (i) had missing or incoherent data on key variables, or (ii) died during treatment or follow-up, or (iii) were diagnosed with relapse before 6 months or later than 36 months post treatment, or (iv) for the first analysis only: relapsed at 6 months.

Individuals who relapsed before 6 months were excluded because they do not contribute to the objectives of this analysis, and those relapsing after 36 months because they are less certainly distinguishable from reinfections.

*Cure* was defined as absence of trypanosomes in all body fluids and < = 20 leucocytes in CSF at ≥22 months post-treatment; and *relapse* as trypanosomes detected in any body fluid or ≥50 CSF leucocytes/µL anytime [7]. Patients diagnosed with relapse without meeting this definition were excluded. We kept the patients who continued on follow-up despite having ≥50 CSF leucocytes/µL and who had a confirmed outcome later (either cure or relapse).

The strict inclusion criteria aimed at strengthening the validity of the results by focusing on patients that provide unequivocal information, using the advantage of having a large cohort.

We defined tolerance windows for each planned follow-up visit: 6 (5–9); 12 (10–16); 18 (17–21); and 24 (≥22) months [2].

Melarsoprol treatment included the following regimens: one series of 10 daily injections; 2 or 3 series of 3 injections; and 3 series of 4 injections. Eflornithine included series of either 7 or 14 days, all at 400 mg/kg/day divided in 4 infusions per day. Combination treatment included melarsoprol-eflornithine, nifurtimox-eflornithine and melarsoprol-nifurtimox co-administrations.

### Statistical analysis

We used the Wilcoxon test to compare CSF leucocytes between different groups of patients. We plotted the evolution of CSF leucocytes (median, IQR) during the follow-up, overall and by treatment received.

#### First analysis

We analyzed the relative change (as a percent reduction) of the CSF leucocytes between baseline (pre-treatment) and 6-months, per patient. We also analyzed the absolute count at 6 months independently of the baseline count. We assessed the accuracy of these 2 diagnostic tests to predict relapse using the receiver-operator-characteristic (ROC) curve and we reported the area under the curve (AUC) with its 95% confidence interval (CI) for each test. For each cut-off of the marker, sensitivity, specificity, positive likelihood ratio (LR+) and negative likelihood ratio (LR−) were reported with their respective 95% CI [8]–[9].

A random-intercept logistic regression was fitted to analyze the effect of the chosen cut-off taking into account several baseline individual characteristics. The threshold p-value to include factors in the initial model was 0.4.

#### Second analysis

following the composite algorithm in two steps at 6 and 12 months proposed by Mumba et al. (at 6 months patients with < = 5 leucocytes/µL are considered cured and with > = 50 leucocytes/µL are considered relapsed, and at 12 months all remaining patients are discriminated with a cut-off at 20 leucocytes/µL) [6], we explored various combinations of cut-off values. The notation we used for the algorithms features the three cut-off values of CSF leukocytes as follows: (i) lower cut-off at 6 months (cure); (ii) upper cut-off at 6 months (relapse), and (iii) unique cut-off at 12 months.

Stata 10 software (StataCorp, College Station, Texas, USA) was used to perform all the data analysis.

## Results

**Figure 1:**
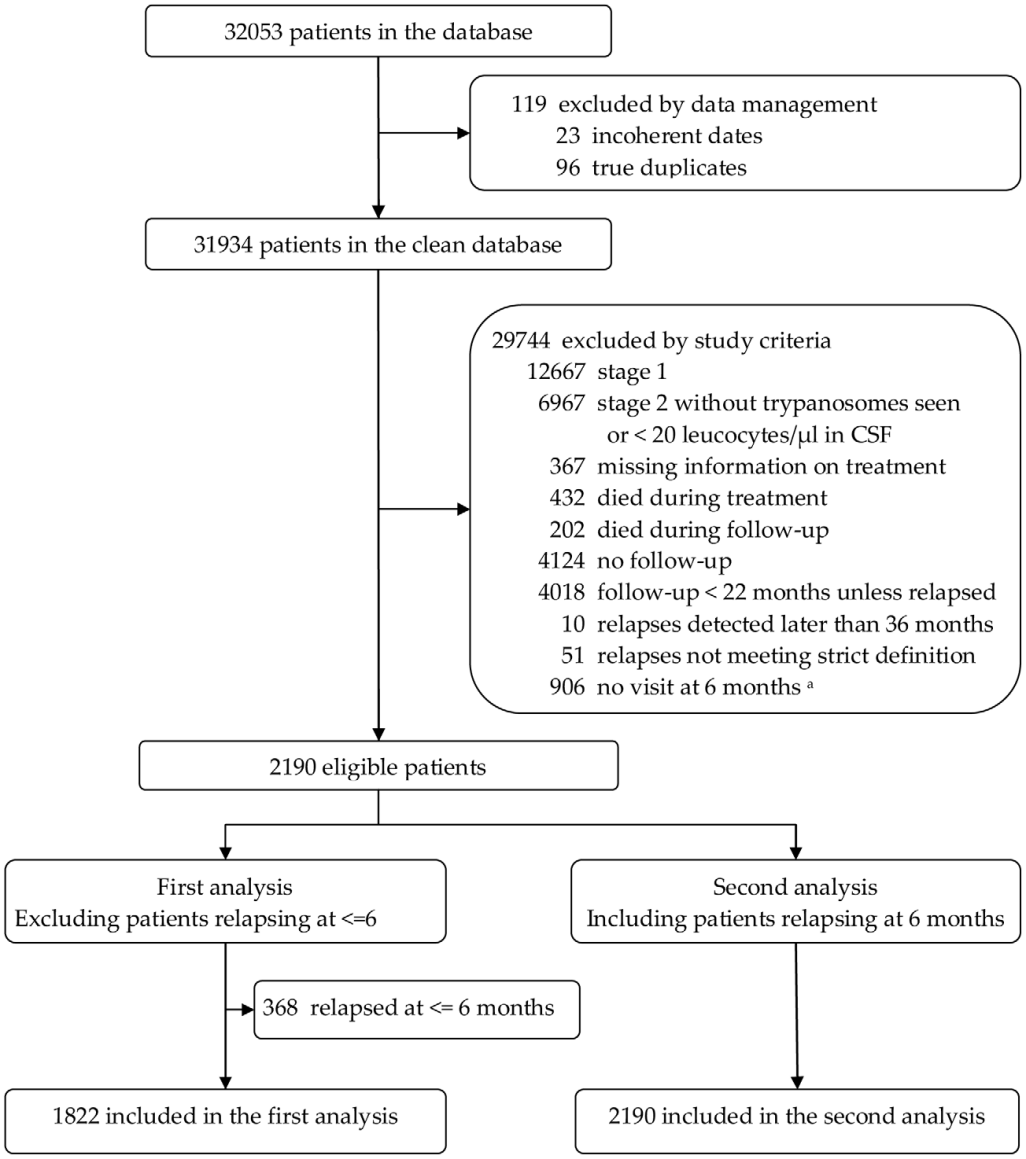
Study profile: selection of study patients. Patients from 12 Médecins Sans Frontières sites in Uganda, Sudan, Angola, Central African Republic, Republic of Congo and Democratic Republic of Congo, 1995–2006. ^a^ Among these 906 exclusions there are 125 patients that relapsed before 6 months (hence falling out of the scope of this study); ^b^ Tolerance window up to 9 months.

Patients selected were 1822 for the first analysis and 2190 for the second analysis (Figure 1) and had been diagnosed between September 1995 and February 2006. Throughout this time period the same diagnostic tools were used. The largest portion of the cohort was from the centers of Omugo, Northern Uganda (44%) and Ibba, Southern Sudan (21%), as these two sites achieved higher follow-up compliance by investing specific resources. Baseline characteristics are shown in Table 1.

**Table 1:** Baseline characteristics of patients included in each analysis.

Characteristics of the patients	First analysis (N = 1822)		Second analysis (N = 2190)	
	N		N	
Sex ratio (M/F)	1821	1.08 (947/874)	2189	1.12 (1159/1030)
Age, median [IQR]	1822	24 [15–35]	2190	24 [15–35]
Weight, median [IQR]	712	49 [9–56]	951	49 [40–56]
Detected by active screening, n (%)	1822	360 (19.8)	2190	405 (18.5)
Trypanosomes detected	1822		2190	
In lymph nodes, n (%)		879 (48.2)		1073 (49.0)
In blood, n (%)		606 (33.2)		712 (32.5)
In CSF, n (%)		1328 (72.9)		1626 (74.3)
CSF leukocyte count	1822		2190	
Mean (SD)		233.2 (326.8)		240.8 (321.5)
Median [IQR]		133 [56–272]		141 [62–287]
20–99 cells/µL n(%)		733 (40.2)		817 (37.3)
≥100 cells/µL n(%)		1089 (59.8)		1373 (62.7)
Clinical characteristics				
Coma score <15, n (%)	626	29 (4.6)	798	34 (4.3)
Karnofsky index, median [IQR]	641	80 [70–80]	831	80 [70–80]
Treatment naivety	1822	1485 (81.5)	2190	1753 (80.0)
Treatment received	1822		2190	
Melarsoprol n(%)		1151 (63.2)		1469 (67.1)
Eflornithine n(%)		540 (29.6)		578 (26.4)
Combinations n(%)		131 (7.2)		143 (6.5)
Patients that relapsed n(%)	1822	362 (19.9)	2190	730 (33.3)

First analysis: leukocytes at 6 months, excluding relapses at 6 months; Second analysis: leukocytes at 6 months, including relapses at 6 months, two-step algorithms. Combination treatment: within this selected cohort, it included melarsoprol-eflornithine, nifurtimox-eflornithine and melarsoprol-nifurtimox combinations. Coma score: Glasgow Coma Scale assessing the level of consciousness. Interpretation: 3–8 = severe impairment; 9–12 = moderate impairment; 13–14 = mild impairment; 15 = normal [13]; Karnofsky index [14].

### Post-therapeutic evolution of the CSF leucocytes count

**Figure 2:**
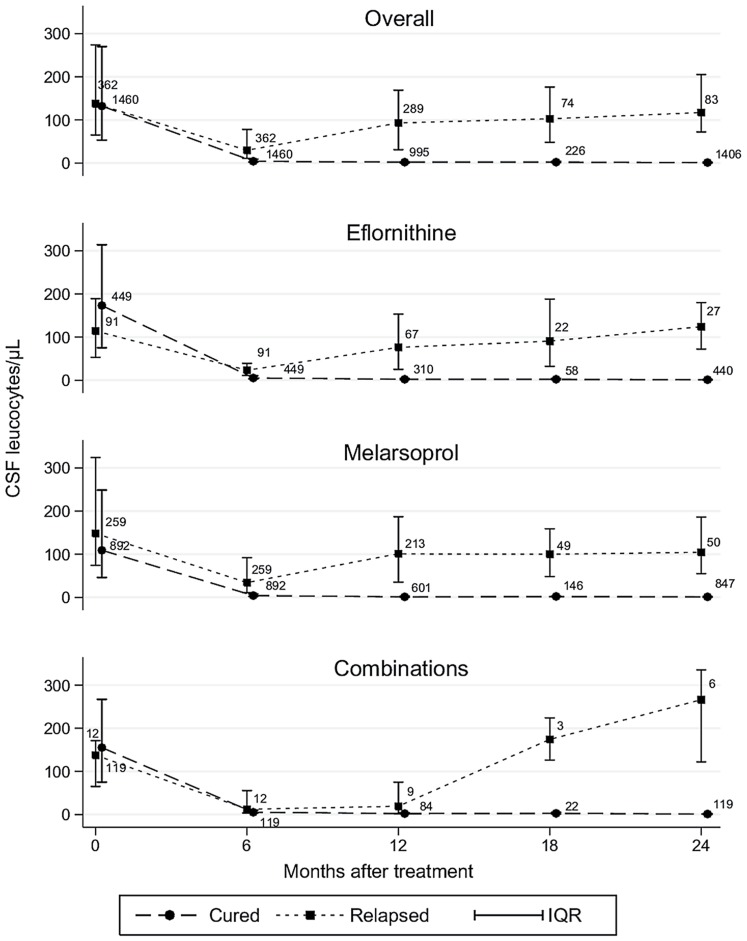
Evolution of the CSF leucocytes count by final treatment outcome, by treatment group and overall. CSF leucocytes count expressed as the median and interquartile range (IQR). Cohort of 1822 patients having a leukocytes count performed at 6 months and not relapsing at 6 months or earlier, who had a complete follow-up. Values at month 0 are pre-treatment measurements. The number of patients per group is shown at each time point. Dotted lines are used to signify that samples over time contain some different patients.

At pre-treatment, the CSF leucocytes count was not different between the 1460 patients who cured (median 137.5 cells, IQR 65–274) and the 362 who later relapsed (132 cells, IQR 53–270) (Wilcoxon test p = 0.15), whereas at 6 months it was significantly higher among patients who later relapsed (29.5 cells, IQR 11–78) than in patients who cured (4 cells, IQR 2–9) (p<0.001). This difference increased at 12 and 18 months, as expected (Figure 2). The difference was observed in all treatment groups, except at 6 and 12 months post-treatment in patients receiving drug combinations, emerging from 18 months onwards.

The evolution of the CSF leucocytes count was similar in naïve (first-time treated) and non-naïve patients throughout the post-therapeutic follow-up (data not shown).

### Prediction of final outcome at 6 months

The ROC analysis showed that the absolute CSF leucocytes count at 6 months was at least as good a predictor of outcome (AUC 0.84) as the percent reduction (AUC 0.81). The latter being also the least practical (requiring a bedside calculation involving the initial laboratory results), we did not explore it further. The CSF leucocytes count at 6 months showed the best trade off between sensitivity and specificity at cut-off values of 10 to 13 leucocytes/µL. The best accuracy was obtained with a cut-off at >10 leucocytes/µL which predicted relapse with 76.2% (95%CI, 71.84–80.65%) sensitivity, 80.4% (95%CI, 78.37–82.45%) specificity, 3.89 (95%CI, 3.45–4.38) LR+, 0.29 (95%CI, 0.26–0.32) LR− (Table 2). The positive predictive value was 0.49 (95%CI, 0.45–0.53) and the negative predictive value 0.93 (95%CI, 0.92–0.94).

**Table 2:** Performance of different cut-off values of CSF leucocytes count at 6 months for the detection of relapse.

Cut-off of CSF leucocytes count at 6 months	Sensitivity	Specificity	Correctly classified	Likelihood Ratio	
				Positive	Negative
>5	83.98%	60.21%	64.93%	2.11	0.27
>10	76.24%	80.41%	79.58%	3.89	0.29
>20	59.67%	93.49%	86.77%	9.17	0.43

The multivariate analysis confirmed, after adjustment on treatment, age and sex, that the six-months CSF leucocytes count, with a cut-off at 10 cells, was very strongly associated with the risk of relapse (odds ratio = 17.2, 95%CI, 12.6–23.5).

### Prediction of final outcome by algorithms at 6 and 12 months

Table 3 shows the performance of the two-steps algorithms when tested with our large dataset (n = 2190) of selected patients with laboratory-confirmed outcome. In the first line we show the results reported by Mumba et al. [6] on a smaller cohort (“algorithm 5-50-20”).

**Table 3:** Comparison of several two-steps (6 and 12 months) algorithms for early outcome determination.

Algorithm	n	Sensitivity	(95%CI)	Specificity	(95%CI)	LR+	(95%CI)	LR−	(95%CI)	False cured	(95%CI)	% classified at 6 months
5-50-20^a^	213	94.4	(86–98)	97.8	(94–100)	42.20	(13.8–129.3)	0.06	(0.02–0.15)	1.9	(0.5–4.9)	66.2
5-50-20^b^	2190	87.4	(85–90)	97.7	(97–98)	37.84	(26.4–54.3)	0.13	(0.11–0.16)	4.5	(3.6–5.5)	66.4
5-20-20	2190	89.4	(87–92)	92.0	(90–93)	11.12	(9.2–13.4)	0.12	(0.09–0.14)	3.8	(3.0–4.7)	74.1
5-20-15	2190	90.0	(88–92)	91.7	(90–93)	10.87	(9.0–13.1)	0.11	(0.09–0.14)	3.6	(2.8–4.5)	74.1
5-20-10	2190	90.1	(88–92)	90.8	(89–92)	9.78	(8.2–11.6)	0.11	(0.09–0.14)	3.5	(2.8–4.4)	74.1
5-30-15	2190	89.4	(87–92)	95.4	(94–97)	19.52	(15.2–25.1)	0.11	(0.09–0.14)	3.8	(3.0–4.7)	70.1
5-40-20	2190	87.7	(85–90)	97.1	(96–98)	29.90	(21.7–41.1)	0.13	(0.10–0.15)	4.4	(3.5–5.4)	67.8
5-40-15	2190	88.7	(86–91)	96.8	(96–98)	27.29	(20.2–36.9)	0.12	(0.10–0.14)	4.0	(3.2–5.0)	67.8
5-40-10	2190	89.5	(87–92)	95.2	(94–96)	18.80	(14.7–24.1)	0.11	(0.09–0.12)	3.7	(2.9–4.7)	67.8

a As reported by Mumba 2010, “algorithm C”: includes deaths as treatment failures and patients with incomplete follow-up;

b Same algorithm tested with our dataset including only laboratory-confirmed outcomes. LR: Likelihood ratio. False cured: fraction of patients that are wrongly classified as cured by the algorithm.

The same algorithm in our dataset predicted relapse with 87.4% sensitivity (95%CI, 85–90), 97.7% specificity (95%CI, 97–98), LR+ of 37.84 (95%CI, 26.4–54.3) and LR− of 0.13 (95%CI, 0.11–0.16). It wrongly classified as cured (false negatives) 87/1945 patients (4.5%; 95%CI, 3.6–5.5). Two thirds (66.4%; 95%CI, 64.4–68.4%) of the patients followed-up were already classified at 6 months.

**Figure 3:**
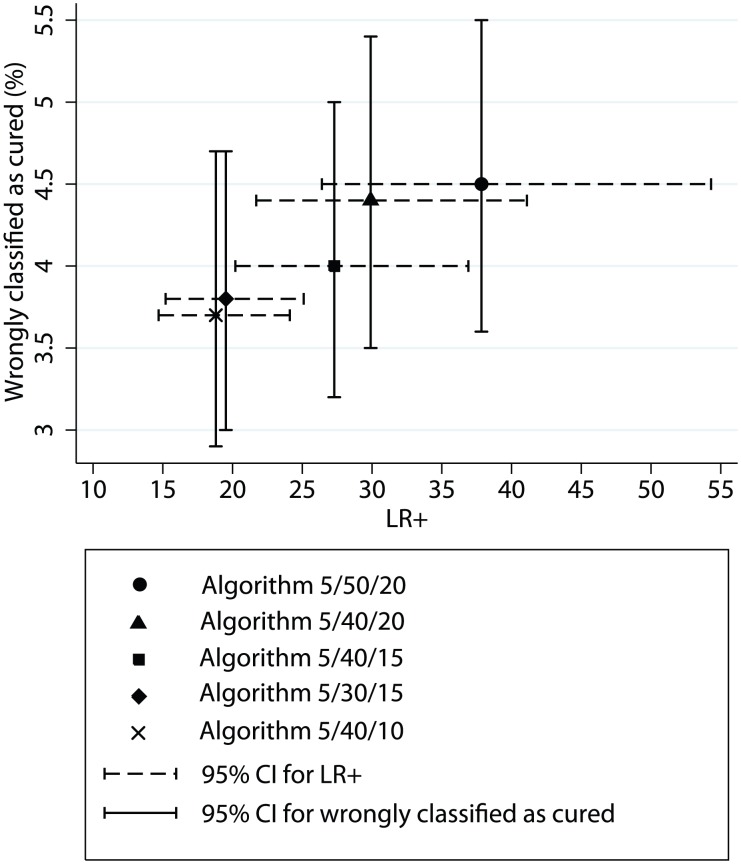
Relationship between the positive likelihood ratio (LR+) and the proportion of patients wrongly classified as cured, for each algorithm tested.

The algorithms 5-40-20 and 5-40-15 also performed well, with confidence intervals overlapping the algorithm 5-50-20. The 5-30-15 algorithm was slightly more sensitive but less specific. The proportion of patients classified as cured who later relapsed ranged from 3.5 to 4.5% in all tested algorithms. The portion of the cohort classified at 6 months ranged from 66.4 to 74.1%, leaving the rest to be classified at 12 months. Of the algorithms tested, the 5-50-20 appeared as the best overall with the highest LR+ and a proportion of false negatives not significantly different from the other algorithms (Figure 3).

## Discussion

The CSF leucocytes count at 6 months showed a good prognostic value for final efficacy outcome. However, a small proportion of patients was wrongly classified. Translated into field patient management, those wrongly classified as relapsed would be unnecessarily re-treated, sometimes with toxic drugs (e.g. melarsoprol if first-line treatment was eflornithine or eflornithine-nifurtimox) and, more importantly, patients wrongly classified as cured would be at risk of death due to HAT relapse. A two-step algorithm, at 6 and 12 months, provided a better classification tool featuring an excellent ability to predict relapses with a lower misclassification rate.

At 6 months, the CSF leucocytes cut-off at 10/µL had the best trade-off between positive and negative likelihood ratios. This indicator can rule out relapse at 6 months post-treatment with a good degree of confidence (0.93 negative predictive value), but its ability to identify true relapses is sub-optimal.

Other cut-off values may be of interest for decision-making in the context of clinical trials, e.g. to continue or suspend enrolment of new participants based on 6-months data (patients already enrolled would always benefit of complete follow up).

It is important to underline that the relapse rate was 19.9% in our dataset (first analysis), which is much higher than the relapse rate reported with the new and increasingly used nifurtimox-eflornithine combination therapy (NECT) [10], [11]. Because positive and negative predictive values depend on the relapse rate, the lower the relapse rate is, the lower will be the positive predictive value for relapse, but on the other hand the negative predictive value will be higher, increasing the confidence on the prediction of cure. Reporting the likelihood ratios allows to make abstraction of this phenomenon, since they are independent from relapse rates. Likelihood ratios, both LR+ and LR− are one of the best ways to measure diagnostic accuracy. In medicine, a test is generally regarded as valuable when the LR+ is >5 or the LR− is <0.2 [9], [12].

Because the CSF leucocytes count at 6 months alone remains insufficiently accurate for outcome determination, we evaluated various two-step algorithms at 6 and 12 months, following the model published by Mumba et al [6].

All tested algorithms performed well, but the 5-50-20 algorithm showed the highest specificity (97.7%) and LR+ (37.8). The sensitivity (87.4%), LR− (0.13) and proportion of patients falsely declared as cured (4.5%) were statistically comparable to the other tested algorithms (table 3, figure 3). Two-third of patients (66.4%) could be classified at 6-months post-treatment. Algorithms that would increase this proportion (such as 5-20-20, 5-20-15 or 5-20-10 that classify 74.1% of patients at 6-months) could be particularly interesting in settings with poor follow-up compliance beyond the first visit at 6 months.

Our findings therefore confirm that the diagnostic algorithm 5-50-20 performs well to predict post-treatment outcome, allowing for a shorter follow-up period.

Other algorithms can be applied depending on the setting and priority objectives, e.g. clinical trials or individual patient management in settings with poor follow-up compliance such as in conflict areas.

In all cases, patients who are declared cured early by using these predictors should be encouraged to come for control if symptoms reappear later.

Early determination of outcome presents several key advantages for HAT control programs: first, it cuts down on uncured patients remaining infective until eventually detected or dying; second, it reduces the workload and costs of follow-up; third, it facilitates the monitoring of treatment effectiveness. For some patients it is life-saving or preventive of serious sequelae, for most others it reduces the burden of complying with follow-up schemes. For clinical studies it accelerates acquisition of results and decreases costs.

### Strengths and weaknesses

One major strength of this study was the restrictive selection criteria, which minimized information bias that is typically present in HAT studies: most cohorts include important proportions of patients with uncertain or unknown efficacy outcome, due to the difficulties in completing the patients' follow-up.

Another strength was the large sample size, which increases the precision of the findings.

Finally, the statistical methods used, in particular the analysis by logistic regression with a random intercept controlling the inter-site heterogeneity.

A weakness arose from the nature of the data used, collected by field routine programs, which is generally of lower quality than data collected prospectively within planned studies.

Another weakness arises from the reference used for “true outcome”: a composite definition based on the presence of trypanosomes or a CSF leucocytes count ≥50. The predictors studied are also based on the CSF leucocytes count (at an earlier time) and are therefore not independent from the outcome measurement. In particular when the predictor includes the same value (CSF leucocytes count ≥50, such as in the algorithm 5-50-20) the specificity is to some extent over-estimated.

The marker at the center of our analysis, the CSF leucocytes count, is subject to measurement error, being a manual laboratory technique. However, this particular laboratory exam is regarded as crucial for the patient and it has been the object of great attention in the MSF sites that were included in this study. Internal quality control was implemented in all field laboratories, through blinded double and triple CSF leucocytes counts, showing good levels of consistency in the results (authors' direct field observation, data not published). To our knowledge there are no published works to shed more light into this issue.

The timing of the follow-up assessments was treated via the consolidation of the visit dates into time “tolerance” windows, which are arbitrary groupings (we followed conventional windows) [2]. This interval censoring is an imperfect way of capturing the timing of events: for example what we treat as the “6 months” leucocytes count in reality happened anywhere between 5 and 9 months, with an uneven spread that tends to concentrate after the 6-months date. This field data distribution can be assumed to correspond well with the reality of the routine programs, but it will fit less the temporal distribution in clinical trials that usually have intensive follow-up of patients.

### Conclusions

This study provides robust evidence on the value of the CSF leucocytes count to predict, at 6 and 12 months, the efficacy outcome of second-stage *T. b. gambiense* HAT treatment.

For decision-making on individual patients followed-up in the field, our findings confirm the good performance of the two-steps algorithm using cut-off values of 5-50-20 leucocytes/µL. Other algorithms can be used depending on the setting.

For the early estimation of efficacy in clinical trials, several options are revealed, both in one step at 6 months and in two steps at 6 and 12 months.
